# CO_2_ Insufflation in Cartilage Repair Using Minced Autologous Cartilage, Platelet-rich Plasma, and Autologous Thrombin: Enhancing Techniques for Repairing Knee Articular Cartilage Defects

**DOI:** 10.1016/j.eats.2025.103835

**Published:** 2025-08-22

**Authors:** Umer Butt, Filip Vuletić, Riccardo Cristiani, Mahenau Afridi, Zainab Aqeel Khan, Yasir Hussain, Anders Stålman

**Affiliations:** a Department of Trauma and Orthopaedics, AO Hospital, Karachi, Pakistan; b Stockholm Sports Trauma Research Center, Department of Molecular Medicine and Surgery, Karolinska Institutet, Stockholm, Sweden; c Department of Arthroscopy and Sports Medicine, Orthopaedic Department Aker, Oslo University, Hospital, Oslo, Norway; d FIFA medical center of excellence, Capio Artro Clinic, Sophiahemmet Private Hospital, Stockholm, Sweden

## Abstract

Injuries to the articular cartilage are challenging to treat because of its limited intrinsic healing capacity, leading to the exploration of various repair methods. The AutoCart technique combines minced autologous cartilage, platelet-rich plasma, and autologous thrombin, with promising clinical results. A key advancement is the use of carbon dioxide (CO_2_) insufflation during arthroscopy, which creates a dry environment and significantly improves the accuracy of defect preparation and graft placement. Unlike traditional fluid distention, CO_2_ insufflation prevents biologically active components of the graft from being washed away, allowing for precise placement and integration within the cartilage defect. Furthermore, it reduces fluid accumulation and leakage into the surrounding soft tissue. In this Technical Note, we describe a technique for performing AutoCart cartilage repair using CO_2_ insufflation, highlighting its advantages over conventional fluid-based methods.

Articular cartilage injuries in the knee remain challenging as a result of the tissue's limited intrinsic healing capacity.^1^ Various repair strategies have been developed, including microfracture, autologous chondrocyte implantation, and osteochondral graft transplantation.^2^ Clinical outcomes appear to be linked to the quality of the repair tissue, with cell-based approaches showing promising results.^3^^,^^4^ In this context, the AutoCart (Arthrex, Munich, Germany) technique has emerged as a potential option.^5^

A single-stage procedure combining minced autologous cartilage, platelet-rich plasma (PRP), and autologous thrombin has been developed to promote cartilage repair and regeneration.^5^ Although evidence remains limited, early in vitro and in vivo studies, along with recent clinical follow-up, suggest encouraging outcomes, including pain reduction and improved activity levels up to 5 years postoperatively.^5^, ^6^, ^7^ Carbon dioxide (CO_2_) insufflation, widely used in laparoscopic surgery because of its rapid absorption and exhalation, has been adapted for knee arthroscopy to create a dry environment and enhance visualization.^8^, ^9^, ^10^ Recent studies suggest that CO_2_ improves joint distraction, reduces surgical time, and facilitates clearer visualization compared with traditional saline distention, without significant complications reported.^10^

In cartilage repair procedures like AutoCart, maintaining a dry environment is crucial for effective graft placement, because it reduces washout of bioactive components.^11^ CO_2_ insufflation may decrease postoperative swelling and discomfort by limiting fluid extravasation.^11^^,^^12^ Although early results are promising, further research is needed to validate the benefits of CO_2_ in knee cartilage repair. This Technical Note outlines the AutoCart technique with CO_2_ insufflation, providing key surgical steps, tips, and common pitfalls for practitioners in arthroscopic cartilage surgery (Video 1).

## Surgical Technique

### Patient Setup, Cartilage Harvesting, and Bone Bed Preparation (Fluid Arthroscopy)

**Fig 1:**
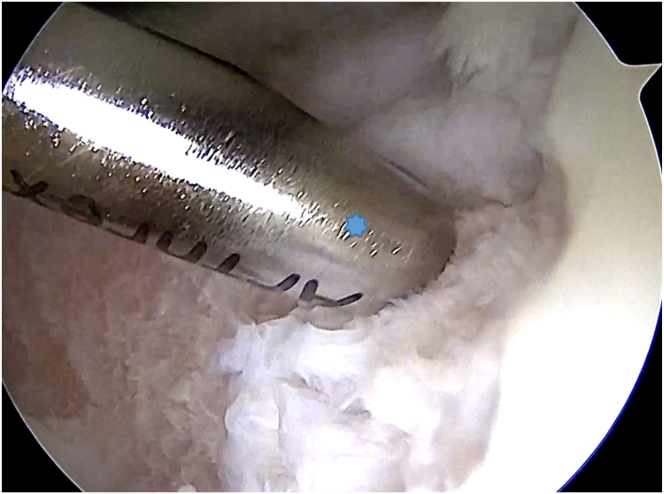
An arthroscopic view of the right knee through the anterolateral portal shows a trochlear defect, with cartilage fragments being removed from its edges using a 3-mm Arthrex Sabre shaver (asterisk) from the anteromedial portal.

The patient is positioned supine, and a high-padded tourniquet is applied. An anterolateral portal is created for diagnostic evaluation, followed by the creation of an anteromedial portal. Hoffa’s fat pad may be resected for better visibility. The cartilage defect is assessed, and unstable cartilage is debrided with a curette. Cartilage fragments are collected using a 3-mm Arthrex Sabre (Arthrex, Munich, Germany) shaver for graft preparation (Fig 1).

**Fig 2:**
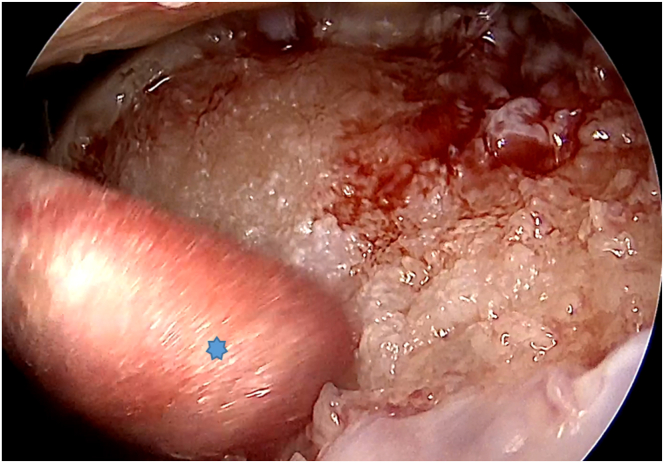
An arthroscopic view of the right knee through the anterolateral portal shows residual moisture being removed with cotton swabs inserted through the anteromedial portal (asterisk).

**Fig 3:**
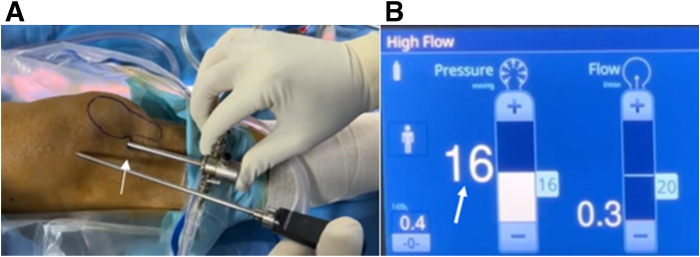
(A) Arthroscope placed in a high anterolateral portal of the right knee (arrow). (B) A modified laparoscopic insufflator fills the knee joint with CO_2_ gas up to a pressure of 20 mm Hg (arrow).

After harvesting the cartilage, the size of the lesion is measured, and the bone bed is prepared using curettes and burrs to create a clean and stable surface while removing any debris or necrotic tissue. Once bone preparation is complete, any saline in the joint is drained, and residual moisture is removed with cotton swabs (Fig 2). CO_2_ insufflation is then initiated through the arthroscope, using a laparoscopic insufflator set at a pressure of up to 20 mm Hg and a flow rate of up to 20 L/min, establishing a dry working environment essential for accurate graft placement (Fig 3).

### Graft Preparation and Application (Dry Arthroscopy)

**Fig 4:**
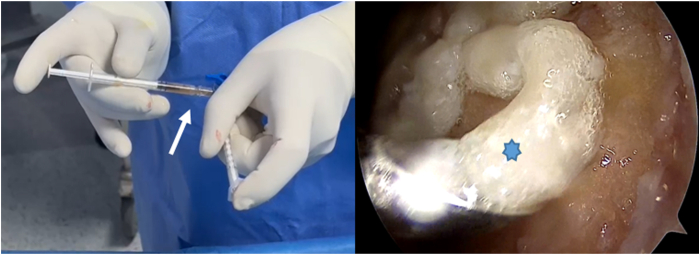
On the left side of the picture, minced cartilage chips are mixed with 0.5 to 1.0 mL of platelet-rich plasma to achieve a cohesive consistency (arrow), using 2 syringes. On the right side, there is an arthroscopic view of the right knee taken through the anteromedial portal, which shows a pasty graft substance (asterisk) applied through the obturator device.

**Fig 5:**
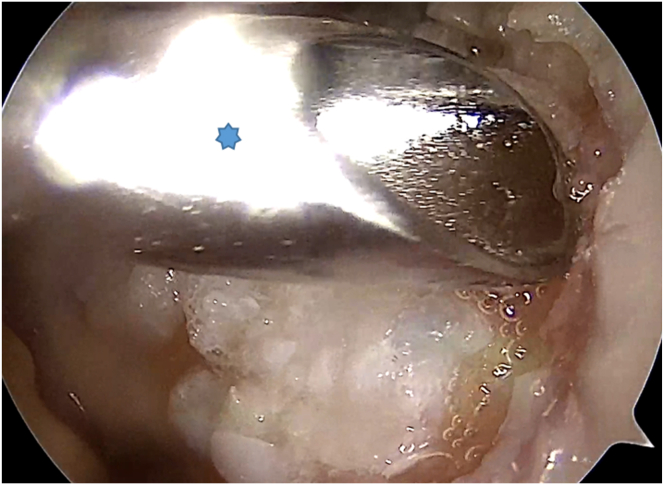
Arthroscopic view of the right knee through the anterolateral portal shows the graft being gently spread with a spatula or obturator device (asterisk) from the anteromedial portal, filling approximately 80% to 90% of the defect height.

Minced cartilage chips are mixed with 0.5 to 1.0 mL of PRP for consistency (Fig 4). Before anesthesia, 10 to 15 mL of blood is drawn from the cubital vein, and 3 mL of PRP is processed with the Thrombinator system (Arthrex) for 10 to 15 minutes to create autologous thrombin. During implantation, autologous thrombin is applied to the cartilage-PRP mixture to enhance fibrin clot formation and stabilize the graft. Optionally, a thin layer of fibrin can be added for sealing. The graft is then spread with a spatula to fill about 80% to 90% of the defect height without needing to match the surrounding cartilage level (Fig 5). Steps of the surgical technique, along with tips and potential pitfalls, are outlined in Table 1.

**Table 1:** Surgical Steps, Pearls, and Pitfalls of CO_2_ Insufflation for Performing the AutoCart

Surgical Step	Pearls	Pitfalls
Portal creation	Use a small stab incision and a hemostat clamp or blunt trocar to puncture the joint capsule. This technique seals soft tissue and reduces gas leakage.	A “loose seal” portal may increase gas leakage.
Placement of cannula and debridement of the Hoffa’s fat pad	Reduces obstruction of the Hoffa’s fat pad and improves the visualization of the chondral defect.	Potential interposition from the Hoffa’s fat pad.
CO_2_ insufflation	The insufflation pump is set at 20 mm Hg.	Nonoptimal pressure settings could impair visualization.
Bony bed preparation	Curettes and shavers create an even surface, which enhances graft placement.	Improper bony bed preparation may lead to poor repair tissue formation.
Graft preparation	Proper graft preparation is essential to create a pastelike substance for uniform distribution and initial stability in the defect area.	Poor graft preparation can make the application more difficult.
Graft application	Filling from 80% to 90% of the surrounding healthy cartilage level is sufficient.^13^	Overstuffing the defect may later lead to graft hypertrophy.

CO_2_, carbon dioxide.

## Discussion

Gas insufflation during the arthroscopic AutoCart cartilage repair procedure offers several benefits but also presents specific limitations (Table 2). Dry arthroscopy enhances visualization and minimizes technical errors, and although CO_2_ was once the preferred method because of its high solubility in blood, it has largely been replaced by saline.^8^, ^9^, ^10^, ^11^, ^12^, ^13^, ^14^ Notably, Riddell^13^ used CO_2_ in more than 3,000 knee arthroscopies without complications, showcasing its effectiveness. In 2006, Coletti et al.^15^ showed its use during matrix-induced autologous chondrocyte implantation and reported no complications linked to CO_2_. In a review, Vascellari et al.^16^ found no complications related to the use of CO_2_ in Coletti et al.’s patient group. Continuous CO_2_ inflation may maintain a stable joint environment, promoting graft integration and reducing fluid leakage into soft tissues.^13^^,^^16^ Earlier studies have shown that dry arthroscopic procedures reduce swelling and accelerate recovery after joint surgeries.^10^^,^^14^^,^^16^ This reduction in swelling can alleviate postoperative pain and promote quicker rehabilitation.^10^ However, it is essential to recognize its limitations and consider the existing literature. The dry arthroscopy method requires a system for fluid irrigation and gas insufflation, necessitating alternating phases for rinsing and the procedure itself.^9^ Current literature indicates no increased systemic risks associated with gas leakage.^10^^,^^16^ It highlights the importance of the surgeon's experience and the need for risk mitigation strategies during the procedure.^10^^,^^13^^,^^15^^,^^16^

**Table 2:** Advantages and Disadvantages of CO_2_ Insufflation for Performing the AutoCart

Advantages	Disadvantages
Dry conditions enable the precise placement of biomaterials without risking the washout of bioactive components.	CO_2_ can cause lens fogging, requiring anti-fogging measures.
It removes floating debris and blood, providing a clearer view of cartilage defects.	Limited experience with CO_2_ insufflation among orthopaedic surgeons.
Less fluid extravasation leads to decreased joint swelling and discomfort after surgery.	Current evidence on long-term outcomes is still scarce.
Minimal risk of gas embolism.	Specialized pumps are required for CO_2_ insufflation and may not be widely available.

CO_2_, carbon dioxide.

In conclusion, CO_2_ insufflation improves visualization and creates favorable conditions for cartilage repair in knee arthroscopy.^15^^,^^16^ Yet, careful management of intra-articular pressure and attention to the learning curve are essential, with further research needed on its long-term effects.

## Disclosures

All authors (U.B., F.V., R.C., M.A., Z.A.K., Y.H., A.S.) declare that they have no known competing financial interests or personal relationships that could have appeared to influence the work reported in this paper.

## Supplementary Data

Video 1 Here, we present the carbon dioxide insufflation in cartilage repair using minced autologous cartilage, platelet-rich plasma (PRP), and autologous thrombin: enhancing techniques for repairing knee articular cartilage defects. The technique can be used for all symptomatic full-thickness cartilage defects in the knee, provided the defect is accessible arthroscopically. Authors Umer Butt and Anders Stalman are consultants for Arthrex. The patient is placed in a supine position, with a high-padded tourniquet applied to the thigh to maintain knee mobility. A standard anterolateral portal is created to allow for single-portal arthroscopic access. Initial arthroscopy is performed through the anterolateral portal for diagnostic evaluation and a thorough inspection of the knee joint. Under direct visualization, a no. 11 blade scalpel and a hemostat clamp or blunt trocar are used to establish the anteromedial portal while minimizing trauma to the capsule and reducing the risk of leakage. If necessary, Hoffa’s fat pad is resected using a shaver to improve visualization. Once the cartilage defect is identified, any unstable cartilage is debrided to reveal stable, vertical edges using a curette. Cartilage fragments are gathered from the edges of the defect with a 3-mm Arthrex Sabre shaver and collected in a GraftNet (Arthrex) vial, which results in minced cartilage suitable for graft preparation. After harvesting the cartilage, the size of the lesion is measured, and the bone bed is prepared using curettes and burrs to create a clean and stable surface while removing any debris or necrotic tissue. Once bone preparation is complete, any saline in the joint is drained, and residual moisture is removed with cotton swabs. Carbon dioxide insufflation is then initiated through the arthroscope, using a laparoscopic insufflator set to 20 mm Hg pressure and a flow rate of 20 L/min, establishing a dry working environment essential for accurate graft placement. Minced cartilage chips are mixed with 0.5 to 1.0 mL of PRP for a cohesive consistency. The graft is gently spread with a spatula to fill approximately 80% to 90% of the defect height, without the need to match the surrounding cartilage level completely. During implantation, autologous thrombin is applied dropwise over the cartilage-PRP mixture to promote fibrin clot formation and stabilize the graft. A thin layer of preprepared fibrin can optionally be added for further sealing.
